# A giant and rapid myocardial remodeling due to fatal giant cell myocarditis: a case report

**DOI:** 10.3389/fcvm.2025.1488503

**Published:** 2025-02-26

**Authors:** Wei Zhang, Tao Guo

**Affiliations:** ^1^ Department of Cardiology, Zhongnan Hospital of Wuhan University, Wuhan, Hubei, China; ^2^ Institute of Myocardial Injury and Repair, Wuhan University, Wuhan, China

**Keywords:** giant cell myocarditis, sudden death, cardiac MRI, heart transplant, early recognition and treatment

## Abstract

Giant cell myocarditis is a rare and rapidly progressive disease with a high mortality rate. We present the case of a 21-year-old male without a medical history who presented with a giant left ventricle (9.9 cm, EF:10%) and in a severe clinical state. Cardiac MRI and virology raised the suspicion of giant cell myocarditis. Concerned about the hemodynamic and respiratory deterioration, we initiated cardiac transplant therapy. A fatal ventricular fibrillation occurs while waiting for the heart transplant. Sudden death could represent the “first symptom” of pathological findings. It is important to recognize that while sudden death due to giant cell myocarditis may be rare, it is still a potentially serious complication of giant cell infection and should be considered in cases of unexplained sudden death. In addition, this case highlights the challenges in the diagnosis and management of giant cell myocarditis and the need for early recognition and aggressive treatment.

## Introduction

Giant cell myocarditis is a rare but potentially fatal condition that can cause severe damage to the heart (1–3.) The clinical presentation of giant cell myocarditis is variable, ranging from asymptomatic or mild symptoms to acute heart failure and sudden cardiac death. The diagnosis of giant cell myocarditis is challenging due to its nonspecific clinical features and the difficulty in detecting the virus in myocardial tissue. In this case report, we present a rare case of fatal giant cell myocarditis in which the patient experienced a rapid and giant myocardial remodeling, leading to heart failure and death. This case highlights the importance of considering giant cell myocarditis as a potential cause of acute heart failure and sudden cardiac death, especially in immunocompromised patients. We also discuss the challenges in diagnosing giant cell myocarditis and the need for prompt recognition and treatment of this condition.

## Clinical presentation

A 21-year-old previously healthy male presented to our hospital with a one-week history of chest tightness and shortness of breath. He reported a recent cold exposure prior to the onset of symptoms. He presented to an outside hospital for evaluation on August 12th, 2022. The chest CT scan shows pulmonary edema, bilateral pleural effusion, pericardial effusion, and cardiomegaly. Treatment with diuretics and bronchodilators has not resulted in improvement.

**Figure 1:**
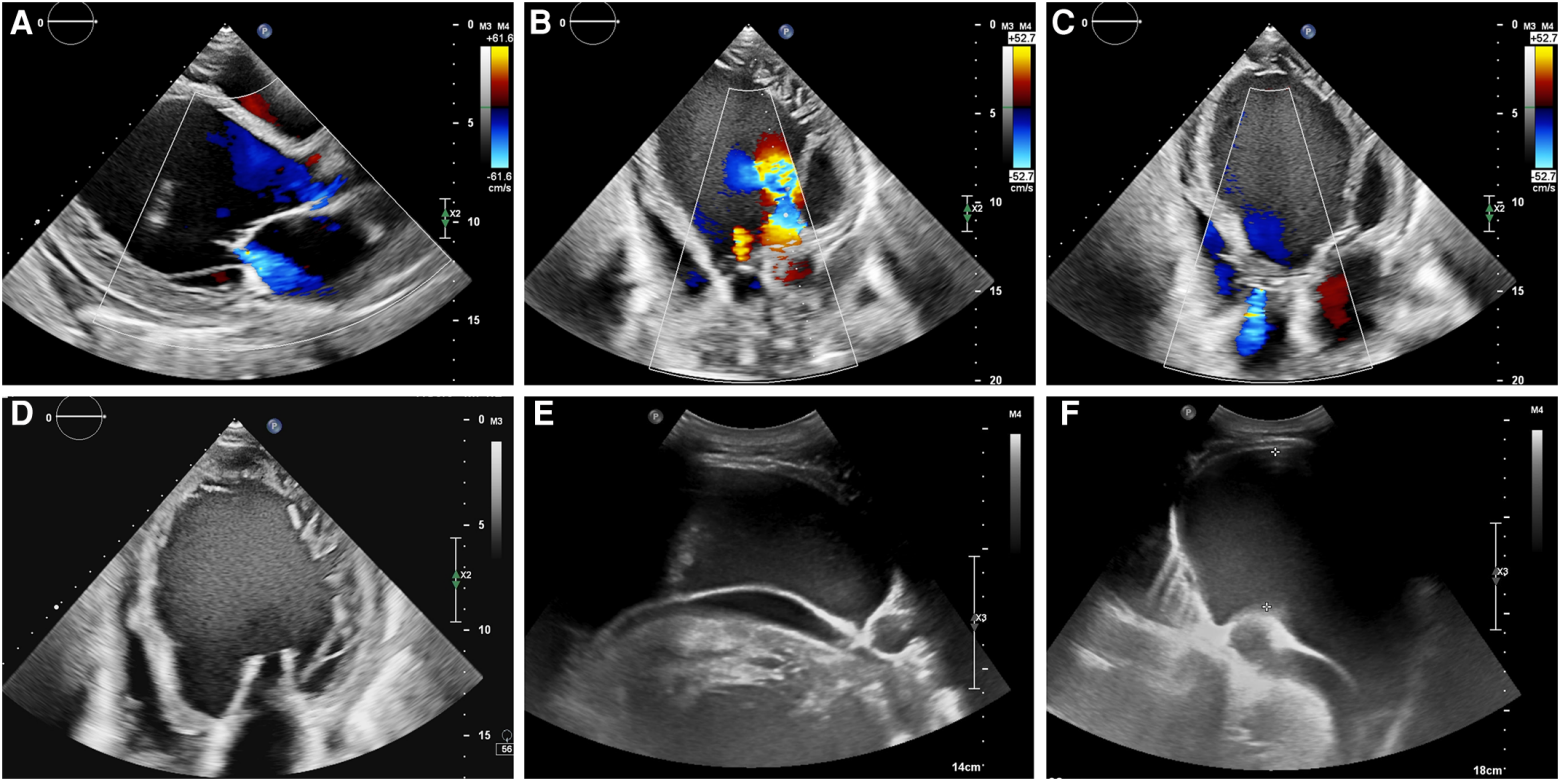
**(A–D)** The echocardiogram showed left ventricular enlargement of 9.9 cm, and left ventricular systolic and diastolic function were decreased, with a small amount of pericardial effusion. **(E,F)** Abdominal ultrasound showed the presence of abdominal fluid accumulation, also known as ascites.

Next day, the patient presented to our outpatient department and an echocardiogram revealed an ejection fraction of 10%, left ventricular enlargement of 9.9 cm (Figure 1), high-sensitivity cardiac troponin level of 9,608 pg/ml, and NT-proBNP level of 19,600 pg/ml. The echocardiogram showed diffuse and significant reduction in left ventricular wall motion, reduced right ventricular wall motion, left ventricular enlargement of 9.9 cm, incomplete local myocardial densification in the left heart, mild aortic valve regurgitation, moderate mitral valve regurgitation, and mild-to-moderate tricuspid valve regurgitation. The pulmonary artery was widened and left ventricular systolic and diastolic function were decreased, with a small amount of pericardial effusion. The electrocardiogram revealed sinus rhythm, abnormal Q waves with ST segment elevation in the inferior and anterior leads, left atrial abnormality, first-degree atrioventricular block, and partial ST-T changes with prolonged QT interval. The COVID-19 nucleic acid test was negative. The patient was subsequently admitted to the Department of Cardiology for further diagnosis and treatment.

The patient's past medical history was had undergone circumcision during childhood. He denied the use of tobacco, alcohol, or illicit drugs. Family history was no family history of sudden death, autoimmune, or connective tissue disease.

On admission, physical examination showed a body temperature of 36.3°C, heart rate of 103 beats per minute, respiratory rate of 20 breaths per minute, blood pressure of 103/67 mmHg, oxygen saturation of 94% on room air, elevated jugular venous pressure, crackles in the lung bases, distant heart sounds, and no edema in both lower limbs.

Laboratory values were notable for the white blood cell count and neutrophil ratio are normal, as are the platelets, monocyte count, and monocyte percentage. However, the D-dimer level is elevated at 2,104 ng/ml, and the high-sensitivity troponin I and NT-proBNP levels are also high at 7,146.8 and 13,300.0 pg/ml, respectively. The creatinine and uric acid levels are elevated at 109.8 umol/L and 1,011.9 umol/L, respectively, and there is positive occult blood in the urine. The alanine aminotransferase level is elevated at 123 U/L, while the total bilirubin level is 43.7 umol/L, the direct bilirubin level is 7.6 umol/L, and the indirect bilirubin level is 36.1 umol/L. The albumin level is 34.2 g/L, and the globulin level is 16.2 g/L. The interleukin-6 level, C-reactive protein level, erythrocyte sedimentation rate, thyroid function, and autoantibody levels are all normal. The patient's IgM level for cytomegalovirus is 0.204, while the IgG level is 166. The ferritin level is 415.81 ng/ml.

**Figure 2:**
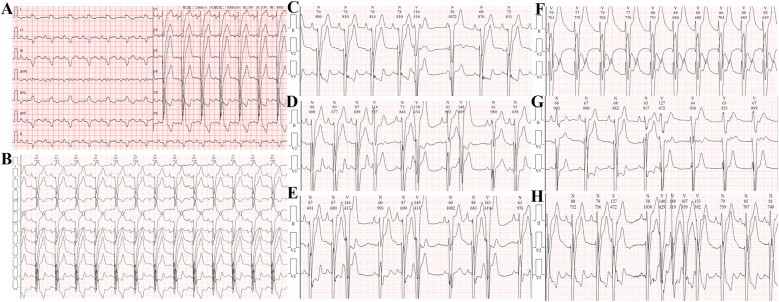
**(A,B)** Electrocardiogram on admission showing ST-segment elevation in leads II, III, aVF, and V1-V4. **(C)** Single ventricular premature beat. **(D)** Couplets of ventricular premature beats. **(E)** Triplets of ventricular premature beats. **(F)** Accelerated idioventricular rhythm. **(G,H)** Ventricular tachycardia.

Electrocardiogram: Sinus rhythm, left atrial enlargement, complete left bundle branch block, PR interval at the critical value, abnormal Q waves with ST segment elevation in the inferior leads, and ST-T changes in some leads with prolonged Q-T interval (Figure 2).

24-hour Holter monitoring revealed a total of 95,559 heartbeats, with the slowest rate of 63 beats per minute, the fastest rate of 125 beats per minute, and an average heart rate of 79 beats per minute. Sinus rhythm with complete left bundle branch block was observed, with 2,148 premature ventricular contractions throughout the recording, including 230 pairs of ventricular premature beats, some of which occurred in runs or trigeminy. Short runs of ventricular tachycardia and accelerated idioventricular rhythm were also recorded, totaling 190 episodes. There were 154 premature atrial contractions throughout the recording. Heart rate variability analysis revealed an SDNN of 48 ms (normal range 102–180 ms) and an SDANN of 70 ms (normal range 92–162 ms). The recording also showed significant ST-T changes.

**Figure 3:**
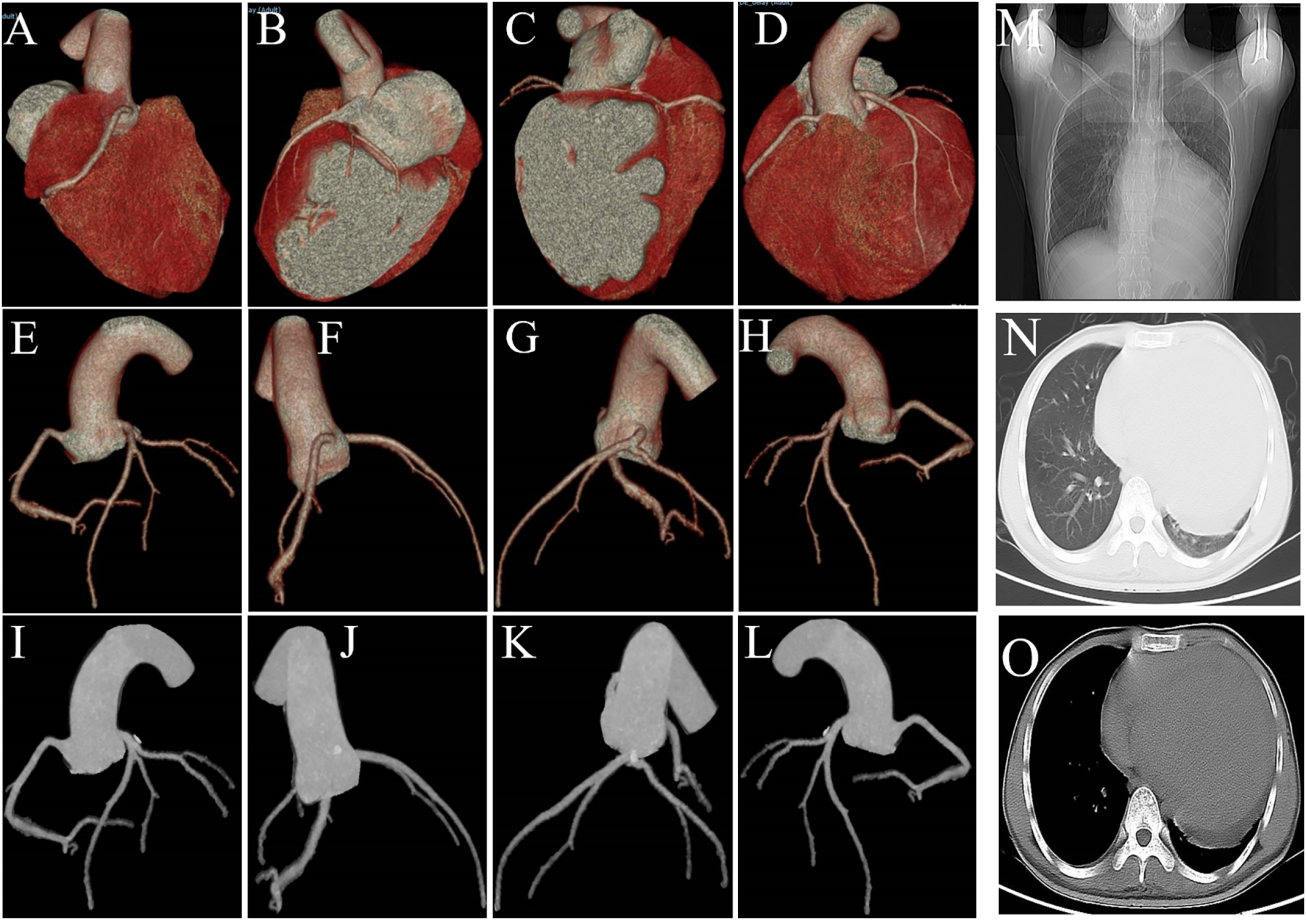
**(A–L)** CT coronary angiography shows no significant stenosis or filling defects. **(M–O)** The CT pulmonary scan reveals inflammation in the lower lobe of the left lung and cardiomegaly.

The CT coronary angiography shows no significant abnormalities in the origins of the left and right coronary arteries, and the coronary arteries do not show any obvious stenosis or filling defects (Figure 3). The patient's heart is right-dominant. The CT pulmonary scan reveals inflammation in the lower lobe of the left lung.

**Figure 4:**
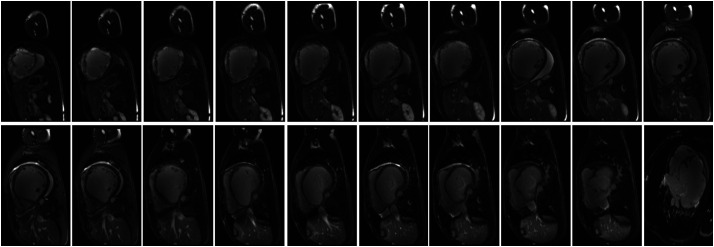
MRI of the heart showed concomitant myocardial inflammation with edema.

MRI of the heart: Multisegmental abnormal enhancement of the left and right ventricular walls, uneven thinning of the interventricular septum with diverticulum formation, enlargement of the left atrium and ventricle, diffuse wall hypokinesis of the ventricle, reduced left ventricular systolic function, and multiple intraventricular mural thrombi. Incomplete myocardial densification of the left ventricle and discordant motion of the interventricular septum, possibly associated with left bundle branch block. Moderate mitral regurgitation, mild aortic regurgitation, and pericardial effusion (Figure 4).

The patient was diagnosed with suspected giant cell myocarditis and started on antiviral therapy (ganciclovir), anticoagulation (unfractionated heparin), RAAS blockade (spirolactone), beta-blockade (metoprolol), ARNI, steroids (methylprednisolone), and supportive care (nutritional support, renal and liver protection). He was listed for heart transplantation and closely monitored in the intensive care unit. He was discussed the possibility of immunosuppressive therapy and the implantable cardioverter-defibrillator (ICD). Despite initial clinical improvement, he developed ventricular fibrillation and was immediately defibrillated. However, he could not be resuscitated despite prolonged advanced life support measures.

## Discussion

Giant cell myocarditis is a rare and fatal condition that can present with nonspecific symptoms and mimic other cardiac diseases. Early recognition and aggressive management are crucial, but the prognosis remains poor even with optimal therapy. This case highlights the need for better understanding of the pathogenesis, diagnosis, and treatment of giant cell myocarditis, and the importance of considering this condition in young adults with unexplained heart failure.

### Epidemiological discussion

Giant cell myocarditis is a rare disease. In the past, autopsy studies in the UK and Japan reported incidence rates of 23.4/100,000 and 6.6/100,000, respectively. However, in recent years, the incidence rate of giant cell myocarditis has decreased due to a reduction in autopsy studies, with some studies reporting an annual incidence rate of around 0.13/100,000 (4).

### Etiologic discussion

The specific etiology of giant cell myocarditis is unclear, but it is currently believed to be related to genetics, infection, and immune factors. Through the combined effects of these factors, immune reactions mediated by T lymphocytes lead to the occurrence of giant cell myocarditis (5).

Viral infections can often trigger lymphocytic myocarditis and subsequently trigger giant cell myocarditis. Reported cases have shown that herpes simplex virus, coxsackievirus B2, and parvovirus B19 can all induce giant cell myocarditis. Due to the similarity in names, giant cell myocarditis is often mistaken for myocarditis caused by cytomegalovirus infection. There have indeed been cases of giant cell myocarditis induced by cytomegalovirus infection, and the combination of the two conditions leads to poor prognosis.

In patients with giant cell myocarditis, nearly 20% have autoimmune diseases, with inflammatory bowel disease, myasthenia gravis, and autoimmune thyroiditis being the most common.

Although the incidence of giant cell myocarditis is similar in men and women (6), male symptoms are often more severe, with faster and more pronounced deterioration of heart function. Studies have shown that this is related to testosterone promoting the progression of giant cell myocarditis.

Genetic abnormalities also increase susceptibility to giant cell myocarditis. Kittleson et al. performed genetic testing on myocardial tissue from patients implanted with left ventricular assist devices due to giant cell myocarditis and found abnormalities in 115 immune regulation and Th1 cell function-related genes (7). Recent studies have found that individuals with genetic abnormalities in the bridging integrator gene have a higher incidence of giant cell myocarditis, which overlaps with the pathogenic gene of arrhythmogenic right ventricular cardiomyopathy.

### Clinical discussion

The most common clinical manifestation of giant cell myocarditis is rapidly progressive heart failure, with some patients presenting with palpitations, syncope, and even sudden death as the initial symptoms. Previous reveals that cases of GCM often present with non-specific symptoms such as fatigue, dyspnea, and chest pain, making diagnosis challenging. Sudden death, as seen in our case, is a rare but recognized complication. In rare cases, as we mentioned earlier, it can present as typical chest pain and be misdiagnosed as myocardial infarction.

Giant cell myocarditis is prone to sustained ventricular tachycardia. It has been reported that nearly 40%–50% of giant cell myocarditis patients can develop sustained ventricular tachycardia (8), which is the main cause of syncope and sudden death. Additionally, conduction block between the atria and ventricles is also not uncommon. When a patient presents with a significant decrease in heart function within a short period (such as days to weeks), along with ventricular tachycardia and/or conduction block (10), or symptoms resembling myocardial infarction with normal coronary angiography, giant cell myocarditis should be considered as a possibility.

### Advanced diagnostic tools

In recent years, advancements in molecular diagnostics and genetic testing have provided new avenues for exploring potential underlying causes of myocarditis, including GCM (9). Molecular diagnostics, such as PCR-based assays, can detect viral genomes directly in myocardial tissue, aiding in the differentiation between viral and idiopathic myocarditis. Genetic testing may identify mutations or variants associated with inflammatory cardiomyopathy, although the role of genetics in GCM specifically remains less well-defined. These diagnostic tools can offer valuable insights into the pathogenesis of GCM and may guide personalized therapeutic strategies in the future.

## Discussion of treatment strategies

It should be emphasized that giant cell myocarditis often progresses rapidly, with significant deterioration of cardiac function within a few days. Therefore, it is strongly recommended that management and treatment be carried out in the intensive care unit (11).

The main manifestation is heart failure, and like other types of heart failure, ACE inhibitors, diuretics, and other anti-heart failure treatments should be used. However, digoxin and beta-blockers should be used with caution during treatment, as they may induce or worsen atrioventricular block.

For patients with concomitant arrhythmias, treatment should be targeted towards the arrhythmia, such as implantation of a pacemaker for high-grade atrioventricular block, use of antiarrhythmic drugs for ventricular tachycardia, or even implantation of an ICD. Due to the complexity and variety of arrhythmias in patients with giant cell myocarditis, careful consideration should be given to the use of pacemakers or ICDs to avoid repeated operations and replacement in the short term. For patients with rapid deterioration of cardiac function and unstable hemodynamics, mechanical assist devices such as ECMO or left ventricular assist devices are needed.

### Prognostic discussion

The fundamental treatment for giant cell myocarditis is to suppress abnormal T-cell immunity, such as the use of steroids, mycophenolate mofetil, and T-lymphocyte monoclonal antibodies, to achieve the goal of delaying or even inhibiting the progression of the disease. For patients who are unresponsive to repeated treatments and have persistent deterioration of cardiac function, cardiac transplantation may be the ultimate option. In the absence of immunosuppressive therapy, the non-transplant survival period of Giant cell myocarditis is approximately 3 months from the onset of symptoms, with almost 100% mortality within 1 year. Currently, with the widespread use of steroids, immunosuppressive agents (12, 13), and T-lymphocyte monoclonal antibodies, the prognosis of Giant cell myocarditis has improved to some extent, with non-transplant survival rates of 69% and 58% at 1 year and 5 years, respectively.

Without treatment, the median survival time is 3 months; while steroid monotherapy only lasts 3.8 months. After combined therapy with azathioprine, it can be extended to 11.5 months, and with cyclosporine, it can be extended to 12.6 months. Typically, giant cell myocarditis can occur after an average of 3 years of heart transplantation. Given that 50% of patients have ventricular tachycardia, pacemaker implantation is often recommended as routine.

Immunosuppressive agents are recommended for long-term use, and there have been reports of Giant cell myocarditis recurrence 8 years after discontinuing immunosuppressive agents. The optimal combination of the aforementioned immunosuppressive agents to achieve the best treatment effect is still under exploration (14)^.^ The 5-year survival rate after heart transplantation for Giant cell myocarditis can reach 71%, which is similar to that for heart transplantation in other conditions. Approximately 20% of patients may experience recurrence of Giant cell myocarditis after heart transplantation, but the response rate to immunosuppressive therapy is relatively high (15).

## Limitations

It is important to acknowledge the limitations inherent in drawing conclusions from a single case report. The findings presented here may not be generalizable to the broader GCM patient population. Additionally, this case does not provide novel insights into the treatment or management of GCM beyond what is already known from existing literature. Further research, including larger cohort studies and clinical trials, is necessary to advance our understanding of GCM and to develop more effective therapeutic interventions.

## Conclusion

Idiopathic giant cell myocarditis is a T lymphocyte-mediated inflammatory response. Although Giant cell myocarditis is rare, it progresses rapidly and has a significant destructive effect on heart function and the cardiac conduction system. Enhanced MRI typically shows delayed enhancement (scar), T2-weighted enhancement signal (myocardial edema). 18FDG-PET shows local filling defects (inflammatory sites), which may help to choose the biopsy site for myocardial biopsy. Early recognition and diagnosis and timely addition of immunosuppressive therapy can delay and improve disease progression.

## Data Availability

The original contributions presented in the study are included in the article/Supplementary Material, further inquiries can be directed to the corresponding author.
